# EEG network reorganization across Alzheimer's disease, frontotemporal dementia, and dementia with Lewy bodies

**DOI:** 10.1002/dad2.70275

**Published:** 2026-02-15

**Authors:** Alberto Benussi, Valentina Cantoni, Federica Palacino, Daniele Altomare, Davide Vito Moretti, Paolo Manganotti, Barbara Borroni

**Affiliations:** ^1^ Neurology Unit, Department of Medical, Surgical and Health Sciences University of Trieste Trieste Italy; ^2^ Neurology Unit, Hospital Care Department of Medicine Azienda Sanitaria Universitaria Giuliano Isontina Trieste Italy; ^3^ Department of Clinical and Experimental Sciences University of Brescia Brescia Italy; ^4^ Competence Centre on Ageing (CCA), Department of Business Economics Health and Social Care (DEASS), University of Applied Sciences and Arts of Southern Switzerland (SUPSI) Manno Switzerland; ^5^ Rehabilitation in Alzheimer's Disease Unit IRCCS Istituto Centro San Giovanni di Dio Fatebenefratelli Brescia Italy; ^6^ Molecular Markers Laboratory IRCCS Istituto Centro San Giovanni di Dio Fatebenefratelli Brescia Italy

**Keywords:** Alzheimer's disease, connectivity, dementia with Lewy bodies, EEG, frontotemporal dementia

## Abstract

**INTRODUCTION:**

Electroencephalography (EEG) provides a temporally precise index of neural dysfunction, capturing changes in oscillatory activity, connectivity, and network organization. While spectral slowing is well documented in Alzheimer's disease (AD), frontotemporal dementia (FTD), and dementia with Lewy bodies (DLB), less is known about how these alterations extend to large‐scale networks.

**METHODS:**

We studied 173 participants: 56 AD, 59 FTD, 26 DLB, and 32 healthy controls (HC). Resting‐state EEG was analyzed to quantify spectral power and amplitude‐envelope correlation‐based connectivity across frequency bands.

**RESULTS:**

AD showed canonical slowing with delta/theta increases and posterior alpha loss. FTD exhibited preserved alpha but frontal beta reductions, while DLB displayed delta/theta excess, posterior alpha attenuation, and uniquely reduced gamma. Connectivity analyses revealed syndrome‐specific patterns of network reorganization with distinct frequency‐dependent signatures.

**DISCUSSION:**

EEG network metrics capture distinct disease signatures and may inform mechanistic models of dementia.

## Highlights

- Electroencephalography (EEG) reveals distinct network alterations in Alzheimer's disease (AD), frontotemporal dementia (FTD), and dementia with Lewy bodies (DLB)
- AD shows theta hypersynchrony and posterior alpha‐beta disconnection
- FTD displays widespread beta hypoconnectivity with focal theta increase
- DLB exhibits global theta hyperconnectivity and posterior hub loss
- Graph metrics capture disease‐specific network reorganization patterns

## 1 BACKGROUND

Neurodegenerative diseases disrupt the brain's temporal dynamics and network architecture long before overt structural atrophy becomes visible on conventional imaging.^1^ Importantly, this disruption is not uniform across syndromes: Alzheimer's disease (AD), frontotemporal dementia (FTD), and dementia with Lewy bodies (DLB) target partially distinct neuronal populations and large‐scale functional systems, reflecting their divergent molecular pathologies and clinical presentations. Electroencephalography (EEG) provides a direct measure of neuronal population activity with millisecond precision and is inherently sensitive to both synaptic dysfunction and network‐level disorganization, making it uniquely positioned to capture syndrome‐specific electrophysiological signatures that complement established biomarkers.

Resting‐state EEG in dementia has consistently demonstrated alterations in oscillatory power and peak frequency, commonly summarized as “spectral slowing” and characterized by increases in delta/theta power with reductions in posterior alpha.^2^ Yet spectral power represents only a summary measure of local oscillatory activity, and mounting evidence indicates that the topography, magnitude, and, critically, the network embedding of this slowing differ substantially across diseases. In AD, parietal‐occipital alpha weakening and increased theta/delta activity are thought to reflect synaptic failure within posterior default‐mode and visual association cortices, regions that show early amyloid deposition and tau accumulation.^3^ FTD preferentially affects anterior salience and executive‐control networks, with network disruption most prominent over frontal and anterior temporal regions that correspond to sites of selective neuronal loss.^4^ In DLB, widespread alpha slowing and impaired posterior rhythms have been linked to thalamocortical dysrhythmia and cholinergic deficits, often paralleling fluctuations in attention and arousal that define this syndrome clinically.^5^ These observations suggest that oscillatory metrics are not merely epiphenomena but windows onto disease‐specific pathophysiology, including synaptic dysfunction, selective network disconnection, and neuromodulatory imbalance, that unfolds at the temporal scale of cognition itself.

Despite these advances, EEG research in dementia remains fragmented in ways that limit translational progress. Studies vary widely in electrode montages, preprocessing pipelines, artefact handling, frequency band definitions, references schemes, and statistical approaches, and most focus on a single dementia syndrome using a single class of metric, typically sensor‐level spectral power. This fragmentation obscures a fundamental question: Does spectral slowing represent a unitary, nonspecific marker of neurodegeneration, or does it embed distinct network‐level signatures that differ systematically across AD, FTD, and DLB? Addressing this requires moving beyond univariate spectral analysis toward a network neuroscience framework that characterizes not only local oscillatory changes but also how brain regions interact and how the topology of these interactions is reorganized by disease.

**RESEARCH IN CONTEXT**

1. **Systematic review**: We searched PubMed and recent conference proceedings for studies examining EEG alterations in Alzheimer's disease (AD), frontotemporal dementia (FTD), and dementia with Lewy bodies (DLB). Prior work has focused primarily on spectral slowing, with fewer studies addressing source‐level connectivity or graph‐theoretical network analysis. No previous study has systematically compared these three major dementia syndromes using a harmonized analytical pipeline.
2. **Interpretation**: Our study demonstrates that electroencephalography (EEG) reveals syndrome‐specific alterations in large‐scale brain dynamics. AD showed theta hypersynchrony with posterior alpha‐beta disconnection, FTD displayed widespread beta hypoconnectivity with focal theta increases, and DLB exhibited global theta hyperconnectivity with posterior hub loss. These results highlight distinct network signatures that reflect underlying disease mechanisms.
3. **Future directions**: Future work should validate these EEG network signatures longitudinally, test their utility for tracking disease progression, and explore integration with molecular biomarkers and clinical outcomes to improve diagnosis and patient stratification.

Among available connectivity metrics, orthogonalized amplitude envelope correlation (AEC) is particularly well suited to resting‐state EEG for several reasons: it captures coordinated fluctuations of regional signal amplitude over timescales of hundreds of milliseconds to seconds,^6^ shows strong correspondence with resting‐state fMRI functional connectivity, and, through signal orthogonalization, effectively mitigates spurious correlations arising from volume conduction and spatial leakage, a critical consideration for source‐reconstructed EEG.^6^ Examining connectivity at the network level provides a principled approach to test whether AD, FTD and DLB express distinct EEG signatures that extend beyond generic spectral slowing.

We therefore undertook a cross‐disease, multiscale EEG study integrating sensor‐level spectral analysis with source‐reconstructed connectivity. Comparing AD, FTD, and DLB within a unified framework is essential because these syndromes target distinct large‐scale networks. By applying identical methods across conditions, we aim to disentangle generic markers of neurodegeneration from syndrome‐specific network signatures, with implications for differential diagnosis, clinical stratification, and mechanistic understanding of how pathology translates into network dysfunction.

## 2 METHODS

### 2.1 Participants

We recruited participants from the University of Brescia memory clinic meeting standard clinical criteria for probable AD, behavioral variant FTD (bvFTD), primary progressive aphasia (PPA), or DLB.^7^, ^8^, ^9^, ^10^ When needed, cerebrospinal fluid (CSF) or amyloid positron emission tomography (PET) confirmed AD pathology. Fluorodeoxyglucose (FDG)‐PET, DaTScan, and genetic testing for monogenic FTD (*GRN*, *C9orf72*, *MAPT*) were used in selected cases, as previously reported.^11^ Age‐matched healthy controls (HCs) with Mini‐Mental State Examination (MMSE) scores ≥ 27 and no psychiatric/neurological illness served as references (see Supplementary Methods). Written consent was obtained (protocol approved by the Brescia Ethics Committee—NP521).

### 2.2 EEG acquisition and processing

Resting‐state, eyes‐closed EEG was recorded for 10 min using a 64‐channel R‐Net system (2500 Hz, Fz reference). Participants were instructed to remain awake with their eyes closed and to avoid movement. Continuous data were visually inspected, and epochs containing gross artefacts or obvious signs of drowsiness (e.g., slow rolling eye movements, muscle relaxation with diffuse high‐amplitude delta) were excluded prior to further processing. Data were preprocessed and cleaned with automated pipelines (EEGLAB, FieldTrip, RELAX, and Discover‐EEG), including filtering, artifact removal, and ICA, retaining comparable artifact‐free epochs across groups.^12^, ^13^, ^14^, ^15^, ^16^ Spectral power (2–50 Hz) was estimated using Welch's method and summarized as relative power, defined as the proportion of total 2–50 Hz power contained in each canonical frequency band (dimensionless, range 0–1). From the regional time series we computed orthogonalized AEC, indexing co‐fluctuations of signal amplitude over slower timescales. Full preprocessing and analysis details are provided in Supplementary Methods.

### 2.3 Statistical analysis

A hierarchical statistical framework was employed to test group differences while controlling for confounders and multiple comparisons. For each electrode (spectral power) or network pair (connectivity), an omnibus permutation‐based one‐way analysis of variance (ANOVA) was first performed to test for group differences (HC, AD, frontotemporal lobar degeneration [FTLD], and DLB) while adjusting for age, sex, and disease severity as covariates. Statistical significance was determined using Freedman–Lane permutation of residuals (5000 permutations), and effect size was quantified using partial eta‐squared (*η* ^2^ *p*). *Post hoc* pairwise comparisons were conducted only for electrodes or network pairs showing a significant omnibus effect (false discovery rate [FDR] ‐corrected *p* < 0.05). Six pairwise contrasts were examined: AD vs HC, FTLD vs HC, DLB vs HC, AD vs FTLD, AD vs DLB, and FTLD vs DLB. Each pairwise comparison used permutation‐based *t*‐tests with 5000 permutations, adjusting for the same covariates using Freedman–Lane permutation of residuals. Multiple comparisons within each frequency band were controlled with Benjamini–Hochberg FDR (*α* = 0.05). Full statistical details are reported in Supplementary Methods.

## 3 RESULTS

### 3.1 Participants

A total of 173 participants were included in the study: 56 AD, 59 FTD, 26 DLB, and 32 HCs. Participants with DLB were significantly older, whereas no significant age differences were observed among HC, AD, and FTD. Educational attainment was significantly lower in DLB compared with HC, FTD, and AD. Disease severity was mild in all patient groups, with median global Clinical Dementia Rating (CDR) scores of 0.5 in AD and DLB, and a median global CDR plus National Alzheimer's Coordinating Center (NACC) FTLD of 0.5 in FTD. As expected, cognitive performance was significantly reduced in all patient groups relative to HC, with no significant differences observed among the patient groups (see Supplementary Table 1).

### 3.2 Sensor‐level spectral power

**FIGURE 1:**
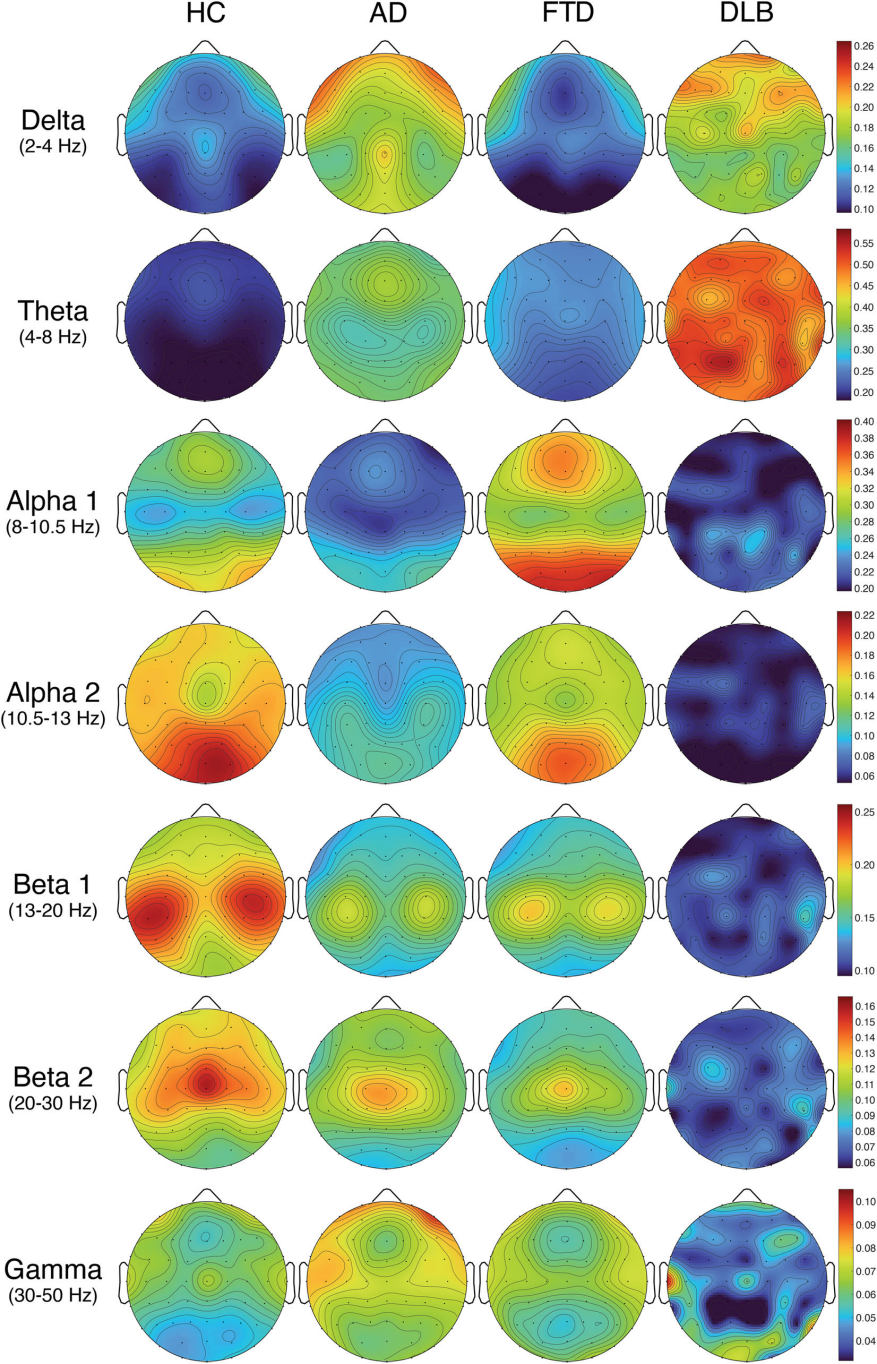
Sensor‐level relative power topographies across groups. Grand‐average scalp maps of relative electroencephalography (EEG) power for healthy controls (HC), Alzheimer's disease (AD), frontotemporal dementia (FTD), and dementia with Lewy bodies (DLB). Rows show canonical frequency bands: delta (2–4 Hz), theta (4–8 Hz), alpha 1 (8–10.5 Hz), alpha 2 (10.5–13 Hz), beta 1 (13–20 Hz), beta 2 (20–30 Hz), and gamma (30–50 Hz). Columns show groups. Color bars indicate relative power values, expressed as the fraction of total 2–50 Hz power (dimensionless), with warm colors indicating higher relative power, cool colors lower power. Color bars show *t*‐values.

**FIGURE 2:**
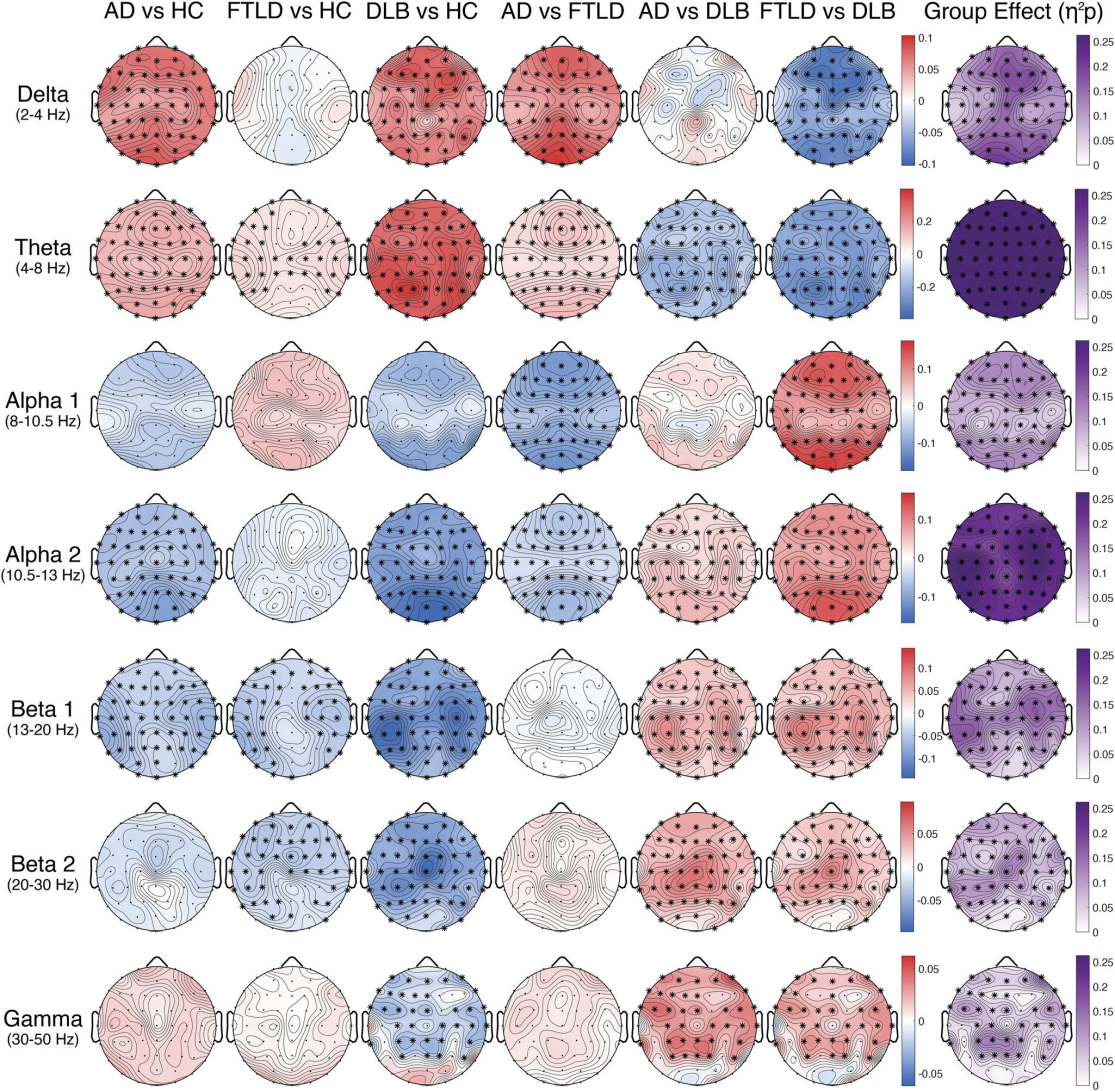
Statistical comparisons of sensor‐level spectral power. Group comparisons of relative electroencephalography (EEG) power using a hierarchical statistical framework. Left six columns show pairwise comparisons: Alzheimer's disease (AD) vs healthy controls (HC), frontotemporal lobar degeneration (FTLD) vs HC, dementia with Lewy bodies (DLB) vs HC, AD vs FTLD, AD vs DLB, and FTLD vs DLB. Rightmost column shows the omnibus group effect quantified as partial eta‐squared (*η* ^2^ *p*). Rows correspond to frequency bands: delta (2–4 Hz), theta (4–8 Hz), alpha 1 (8–10.5 Hz), alpha 2 (10.5–13 Hz), beta 1 (13–20 Hz), beta 2 (20–30 Hz), and gamma (30–50 Hz). For pairwise comparisons, color encodes effect direction (*t*‐values from permutation‐based *t*‐tests with covariate adjustment; blue‐white‐red scale): red indicates higher power in the first group, blue indicates lower power. Asterisks mark electrodes with false discovery rate (FDR) ‐significant differences (Benjamini–Hochberg, *q *< 0.05). *Post hoc* pairwise tests were conducted only at electrodes showing significant omnibus effects. For the omnibus column, color encodes effect size (*η* ^2^; white‐purple scale), with darker purple indicating larger group effects.

Relative power spectra revealed distinct group‐specific alterations across frequency bands. Omnibus testing identified significant group effects at multiple electrodes across all frequency bands, with effect sizes (*η* ^2^) indicating medium‐to‐large differences, particularly pronounced in the theta and alpha ranges (see Figures 1 and 2).

In AD, a clear pattern of spectral slowing emerged. Both delta and theta power were significantly increased relative to controls, with omnibus effects revealing widespread involvement across central, temporal, and parietal electrodes. *Post hoc* comparisons confirmed significant AD versus HC differences with elevated slow‐frequency activity diffusely distributed across the scalp. Alpha power was significantly reduced, particularly over posterior electrodes, whereas beta power showed modest reductions primarily in beta‐1. Gamma activity did not differ significantly from controls (see Figures 1 and 2).

In FTLD, spectral alterations were more circumscribed. Delta power did not differ significantly from controls, while theta power showed a mild increase, predominantly over frontotemporal regions. Critically, alpha activity was largely preserved compared to HC, distinguishing FTLD from both AD and DLB. In contrast, beta power was significantly reduced, particularly over frontal and temporal electrodes. Gamma power was not significantly altered. Direct comparisons between AD and FTLD revealed significant differences in theta (higher in AD) and alpha (lower in AD), reflecting the more prominent posterior involvement in AD (see Figures 1 and 2).

In DLB, spectral changes were the most pronounced across all groups. Delta and theta power were markedly elevated, exceeding the increases observed in AD, with widespread topographic distribution. Alpha power was strongly reduced across all posterior electrodes, reflecting profound weakening of the posterior alpha rhythm. Beta power was significantly diminished across both beta‐1 and beta‐2 sub‐bands. Importantly, gamma power was also reduced in DLB, a finding not observed in the other dementia groups, with significant reductions evident over posterior electrodes. *Post hoc* comparisons confirmed that DLB differed significantly from both AD and FTLD in multiple frequency bands, with theta hyperactivity and alpha‐beta hypoactivity being particularly prominent (see Figures 1 and 2).

Relative power profiles in the FTD subgroups showed selective alterations that differed between the PPAs and bvFTD. In PPAs, spectral alterations were most evident over the frontotemporal regions. Patients exhibited a significant increase in theta power, particularly localized to the left frontotemporal cortex, accompanied by a reduction in beta‐1 power across the same regions. In bvFTD, the spectral profile was more widespread. Patients demonstrated a significant increase in both theta and alpha‐1 power, coupled with a reduction in alpha‐2 power in temporal regions. In addition, beta power was consistently reduced, extending across multiple cortical regions (see Supplementary Figure 1).

### 3.3 Source‐space connectivity

**FIGURE 3:**
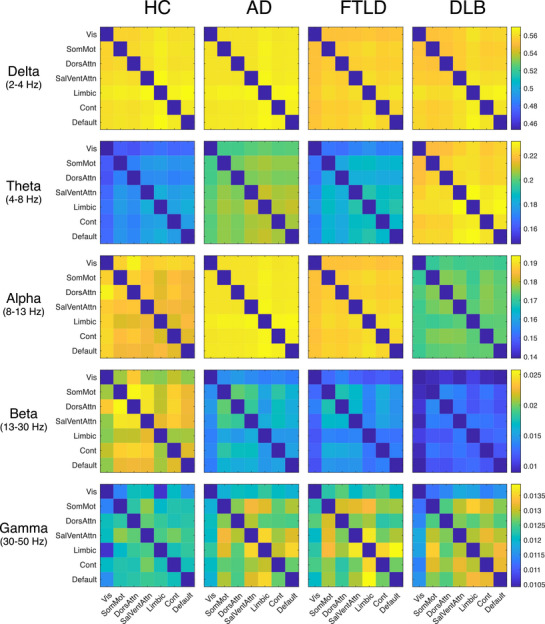
Network‐level amplitude‐based connectivity (AEC) across groups. Group‐averaged 7 × 7 connectivity matrices showing between‐network AEC. Source‐level AEC was aggregated to the Schaefer‐2018 seven‐network parcellation: Vis = Visual, SomMot = Somato‐Motor, DorsAttn = Dorsal Attention, SalVentAttn = Salience/Ventral Attention, Limbic, Cont = Control, Default. Rows show frequency bands: delta (2–4 Hz), theta (4–8 Hz), alpha (8–13 Hz), beta (13–30 Hz), and gamma (30–50 Hz). Color bars indicate AEC values, with warm colors (yellow) indicating stronger connectivity and cool colors (blue) indicating weaker connectivity.

**FIGURE 4:**
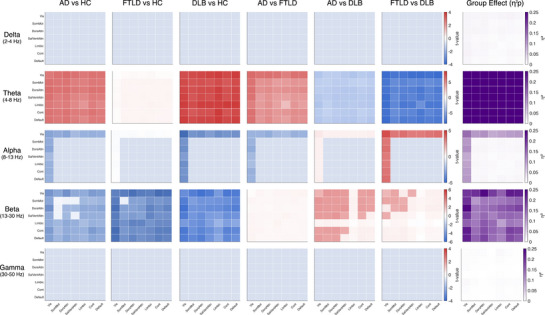
Statistical comparisons of network‐level connectivity. Group comparisons of source‐level amplitude‐based connectivity (AEC) using a hierarchical statistical framework. Left six columns show pairwise comparisons: Alzheimer's disease (AD) vs healthy controls (HC), frontotemporal lobar degeneration (FTLD) vs HC, dementia with Lewy bodies (DLB) vs HC, AD vs FTLD, AD vs DLB, and FTLD vs DLB. Rightmost column shows the omnibus group effect quantified as partial eta‐squared (*η* ^2^ *p*). Rows correspond to frequency bands: delta (2–4 Hz), theta (4–8 Hz), alpha (8–13 Hz), beta (13–30 Hz), and gamma (30–50 Hz). Each 7 × 7 matrix summarizes connectivity between network pairs (Vis = Visual, SomMot = Somato‐Motor, DorsAttn = Dorsal Attention, SalVentAttn = Salience/Ventral Attention, Limbic, Cont = Control, Default). For pairwise comparisons, color encodes effect direction (*t*‐values; blue‐white‐red scale): Red indicates higher AEC, blue indicates lower AEC. *Post hoc* pairwise tests were conducted only at network pairs showing significant omnibus effects. For the omnibus column, color encodes effect size (*η* ^2^ *p*; white‐purple scale). Saturated colors indicate false discovery rate (FDR) ‐significant effects (Benjamini–Hochberg, *q* < 0.05); pale colors indicate nonsignificant differences.

Network‐level amplitude‐based connectivity (AEC) revealed distinct, frequency‐specific alterations across the three patient groups compared with HC. Omnibus testing across the seven canonical functional networks identified significant group effects predominantly in theta, alpha, and beta bands, with effect sizes indicating medium‐to‐large differences (see Figures 3 and 4).

In AD, the connectivity profile was dominated by global theta hyperconnectivity accompanied by distributed alpha and beta hypoconnectivity. Theta elevations were strong and widespread, with significant increases evident in coupling between dorsal attention and visual, salience/ventral attention, control, default, and somatomotor networks. *Post hoc* comparisons confirmed that AD showed significantly higher theta connectivity than HC across nearly all network pairs. By contrast, alpha and beta reductions were most pronounced in posterior and long‐range connections, particularly involving visual, default, and control networks. Delta connectivity remained largely unchanged relative to HC, while gamma showed only modest, nonsignificant changes (see Figures 3 and 4).

In FTLD, a dual pattern emerged characterized by diffuse theta hyperconnectivity and widespread beta hypoconnectivity. Theta increases were prominent across frontoparietal connections and within salience/control networks, though less extensive than in AD. The most striking FTLD signature was in beta connectivity, where significant reductions were broadly distributed across nearly all network pairs. Alpha connectivity was essentially unaffected in FTLD, representing a key distinguishing feature from both AD and DLB (see Figures 3 and 4).

DLB exhibited the most pronounced connectivity alterations across all groups. Theta hyperconnectivity was striking and extensive, affecting virtually all network pairs including extensive posterior‐anterior couplings, with the largest effect sizes observed in comparisons with HC. This was accompanied by marked alpha and beta hypoconnectivity, particularly within the visual network and its long‐range connections to dorsal attention, control, and default networks. *Post hoc* comparisons between DLB and the other patient groups revealed that DLB showed significantly greater theta hyperconnectivity than both AD and FTLD, alongside significantly greater alpha hypoconnectivity. The combination of slow‐frequency hypersynchrony with pronounced fast‐frequency disconnection was unique to DLB (see Figures 3 and 4).

## 4 DISCUSSION

The present study provides a characterization of EEG abnormalities across AD, FTLD, and DLB, integrating sensor‐level spectral power with source‐level network connectivity within a common statistical framework. Beyond confirming that spectral slowing and network changes occur in all three disorders, our data reveal syndrome‐specific patterns that can be interpreted in light of the distinct distribution of pathology, neurotransmitter changes, and clinical features in these three major dementia syndromes.

At the sensor level, AD showed the expected spectral slowing, with increased theta‐delta power and attenuation of posterior alpha and beta rhythms.^17^ This profile is consistent with selective vulnerability of posterior default‐mode and multimodal association cortices, where amyloid‐*β* and tau pathology accumulate early and disrupt thalamocortical loops that support fast oscillations.^18^, ^19^, ^20^, ^21^, ^22^, ^23^ The posterior topography of alpha and beta attenuation mirrors patterns of hypometabolism and atrophy on FDG‐PET and MRI and provides an electrophysiological correlate of episodic memory and visuospatial deficits. By contrast, FTLD showed relatively mild global slowing with preservation of posterior alpha, but more prominent changes over frontal and anterior temporal electrodes, in keeping with predominant involvement of salience, executive, and language networks.^24^, ^25^, ^26^ Within the FTLD spectrum, behavioral‐variant FTD displayed widespread theta and alpha‐1 changes over frontotemporal sites, compatible with degeneration of networks mediating social cognition and behavioral regulation,^27^, ^28^, ^29^ whereas primary progressive aphasia showed increased theta power and focal beta reductions over left perisylvian regions, in line with breakdown of dorsal language pathways.^30^ DLB displayed the most extensive slowing, with diffuse theta–delta increases and marked alpha attenuation over posterior sensors together with gamma‐band reductions over occipital sites. This combination of slow‐band dominance and loss of fast rhythms is congruent with thalamocortical dysrhythmia and cholinergic and occipital dysfunction in DLB, and may underlie the characteristic fluctuations of attention and visuoperceptual disturbances that define this syndrome clinically.^5^, ^23^, ^31^, ^32^, ^33^, ^34^

Connectivity analyses further demonstrated that the three syndromes differ not only in where oscillatory power is altered, but also in how large‐scale interactions between networks are reorganized. In AD, amplitude‐based connectivity revealed reduced alpha and beta coupling within posterior default‐mode and frontoparietal networks, with relatively increased slow‐band coupling.^35^, ^36^, ^37^ This pattern suggests that loss of fast long‐range interactions among posterior networks is accompanied by increased slower, more locally synchronized activity, possibly reflecting compensatory, or pathological reorganization. In FTLD, connectivity changes were more anteriorly weighted, with altered beta interactions among frontal, anterior temporal, salience, and executive‐control networks, compatible with impaired top‐down control and behavioral regulation.^38^, ^39^ Notably, alpha and theta connectivity was largely preserved in FTLD, providing a potential distinguishing feature from AD. In DLB, connectivity revealed marked slow‐frequency hypersynchrony together with pronounced alpha‐beta hypoconnectivity, particularly within visual networks and their long‐range connections to parietal and frontal cortices. This pattern accords with thalamocortical dysrhythmia models of Lewy body disease and offers a plausible network mechanism for the combination of visual hallucinations, cognitive slowing, and attentional fluctuations that characterize DLB.^40^

Several limitations should be acknowledged. First, we analyzed only eyes‐closed resting‐state EEG. Although recordings were obtained in a controlled environment, vigilance was monitored at the bedside and epochs with obvious drowsiness or gross artefacts were rejected, brief fluctuations in arousal cannot be excluded, particularly in patients with dementia, and may have contributed to some slow‐frequency changes. Second, the eyes‐closed condition likely reduced sensitivity to gamma‐band effects; gamma power and connectivity were analyzed but should be interpreted cautiously, and task‐based or eyes‐open paradigms may reveal additional gamma alterations, especially within default‐mode and visual networks. Third, the sample sizes, although relatively large for an EEG study of this kind, were uneven across groups, with fewer patients in the DLB cohort, which may have influenced the stability and generalizability of some DLB‐specific estimates. Fourth, patients were matched for disease severity using global CDR indices, but subtle clinical heterogeneity, including variable cognitive and behavioral phenotypes within FTD and comorbidities, may have introduced additional variance in the electrophysiological patterns. Finally, while source reconstruction and parcellation were applied to mitigate volume conduction and improve anatomical specificity, residual confounds cannot be fully excluded, and EEG's relatively limited spatial resolution compared to magnetoencephalography (MEG) or multimodal imaging constrains inferences about subcortical contributions. Future studies integrating EEG with structural, metabolic and molecular biomarkers, and extending to task‐based and eyes‐open paradigms, will be essential to fully position these electrophysiological signatures within the broader pathophysiological landscape of neurodegeneration.

These findings carry several clinical implications. First, the distinct EEG signatures may aid differential diagnosis when clinical presentations overlap, as frequently occurs in early disease stages: the presence of preserved posterior alpha with frontal beta disruption would favor FTLD over AD, whereas gamma attenuation and widespread theta hyperconnectivity would support DLB. Second, the network‐level characterization could inform patient stratification for clinical trials: interventions targeting posterior default‐mode dysfunction may be most appropriate for AD, whereas trials addressing frontal executive networks may be better suited to FTLD, and treatments aimed at thalamocortical or cholinergic systems may preferentially benefit DLB. Moreover, the frequency‐specific nature of these signatures could guide the design of noninvasive brain stimulation protocols using transcranial alternating current stimulation (tACS) to entrain or restore disrupted oscillatory activity, an approach that has shown promise in modulating cognition in both healthy individuals and patients with neurodegeneration.^41^, ^42^, ^43^, ^44^, ^45^ Third, EEG connectivity measures could serve as pharmacodynamic biomarkers: the theta hyperconnectivity and alpha hypoconnectivity observed in DLB, for example, might be expected to normalize with treatment, providing an objective marker of therapeutic response that complements clinical ratings. Importantly, all patients in our cohort had mild disease severity, demonstrating that these signatures are detectable early, when diagnostic uncertainty is greatest, and therapeutic decisions most consequential.

In conclusion, our findings demonstrate that AD, FTLD, and DLB exhibit distinct electrophysiological signatures that are identifiable at the mild disease stage using a standardized analytical framework combining spectral power and network connectivity. The convergence of these EEG patterns with abnormalities detected by FDG‐PET, resting‐state fMRI, and structural connectivity studies supports their biological validity, while their detection with a widely accessible, noninvasive, and inexpensive modality supports their clinical feasibility. As the field moves toward network‐based biomarkers that complement molecular diagnostics, EEG connectivity analysis could offer a practical tool for differential diagnosis when clinical phenotypes overlap, for stratifying patients into mechanism‐based therapeutic trials, and for monitoring treatment response in syndromes where disease‐modifying and symptomatic interventions are increasingly available.

## CONFLICT OF INTEREST STATEMENT

A.B. has received speaker honoraria from Angelini Pharma, Eli Lilly, Novo Nordisk and has served at scientific advisory boards for Eisai and Eli Lilly; he received research grants from Airalzh, Fondazione Cariplo, the Fondation pour la Recherche sur Alzheimer, and the Italian Ministry of University & Research; he is listed as an inventor on issued patents on the use of noninvasive brain stimulation for the differential diagnosis of dementia and to increase cognitive functions in patients with neurodegenerative disorders. B.B. has served at scientific advisory boards for Alector, Alexion/Astrazeneca, AviadoBio, Lilly, Denali, Wave, and UCB; she is listed as an inventor on issued patents on the use of noninvasive brain stimulation for the differential diagnosis of dementia and to increase cognitive functions in patients with neurodegenerative disorders. V.C., F.P., D.A., D.V.M., and P.M. have nothing to disclose. Author disclosures are available in the supporting information.

## CONSENT STATEMENT

Full written informed consent was obtained from all subjects according to the Declaration of Helsinki. The study protocol was approved by the Brescia Ethics Committee (NP521).

## Supporting information



Supporting Information

Supporting Information
