# Supplementary material for: EEG network reorganization across Alzheimer's disease, frontotemporal dementia, and dementia with Lewy bodies
Source: Alzheimers Dement (Amst). 2026 Feb 15;18(1):e70275. doi: 10.1002/dad2.70275 (PMC12906648; doi:10.1002/dad2.70275)
Supplement: Supplementary file 1 — Supporting Information [file DAD2-18-e70275-s002.docx]

**Methods**

***Participants***

This cohort study involved participants recruited from the Centre for Neurological Disorders at the University of Brescia, Italy. The included participants met the current clinical criteria for probable AD [1], a frontotemporal lobar degeneration-associated syndrome, including behavioural variant of frontotemporal dementia (bvFTD) [2] and the primary progressive aphasias (PPA) [3], and DLB [4]. All participants underwent an extensive neuropsychological evaluation, following standard procedures, as previously reported [5]. Disease severity was assessed with the clinical dementia rating (CDR) [6] or with the CDR plus National Alzheimer's Coordinating Centre (NACC) behaviour and language domains (CDR plus NACC FTLD) global score [7].

Brain MRI scans were performed on all participants. For a subgroup of participants, the diagnosis was confirmed with amyloid markers, including CSF total tau, p-tau_181_, and amyloid-β_1-42_ determinations, or amyloid PET imaging with [^18^F]-florbetapir or [^18^F]-flutemetamol, either supporting or ruling out an AD diagnosis, as previously reported [5]. When diagnostic confidence in selected cases was not satisfactory, additional procedures, including brain FDG-PET and SPECT DaTScan, were employed. Furthermore, in familial cases (defined by the presence of at least one dementia case among the first-degree relatives) and early onset sporadic cases, genetic screening for monogenic forms of FTD, such as *GRN*, *C9orf72* and *MAPT* variants, was performed.

Moreover, a group of age-matched healthy controls (HC) was also enrolled as reference group. HCs underwent a brief standardized neuropsychological assessment, with a mini-mental state examination (MMSE) score of ≥27/30 required for inclusion. Psychiatric or other neurological illnesses were considered exclusion criteria.

Full written informed consent was obtained from all subjects according to the Declaration of Helsinki. The study protocol was approved by the Brescia Ethics Committee (NP521).

## ***EEG recordings***

In a quiet, temperature- and light-controlled room, resting-state, eyes-closed EEG was recorded with an R-Net 64-channel electrode system connected to an actiCHamp Plus 64 System amplifier (Brain Products, Gilching, Germany). A single cephalic ground electrode and a referential montage with Fz as the physical reference were used in line with IFCN standards. Signals were digitized at 2,500 Hz with 24-bit resolution; no filters were applied during acquisition. Vigilance was monitored by visual inspection of the traces; signs of drowsiness (e.g., slowing, slow eye movements, sleep spindles and/or K-complexes) prompted reminders to remain alert. Resting-state EEG duration was 10 minutes (eyes closed). Despite these procedures, brief subclinical fluctuations in vigilance cannot be completely excluded and are considered in the limitations of the study. All artifact detection and quantitative analyses were performed offline.

## ***EEG analysis***

Preprocessing was performed in MATLAB R2024b (The MathWorks, Natick, MA, USA) using EEGLAB [8], FieldTrip [9], the Brain Connectivity Toolbox [10], the RELAX pipeline [11], and custom scripts adapted from the Discover-EEG pipeline [12]. Continuous EEG was first band-pass filtered from 1-80 Hz using a fourth-order zero-phase Butterworth filter to remove slow drifts and high-frequency noise. 50 Hz line noise was removed using Zapline-Plus, which adaptively identifies and removes narrow-band peaks (including harmonics) after segmenting data into spatially stable chunks while preserving spectral content and data rank [13]. Noisy channels were detected via flatline duration, low correlation, and excessive line noise (PREP criteria) [14], removed, and then reconstructed by spherical-spline interpolation to restore the full 64-channel montage. Extreme non-recoverable artifacts were detected by segmenting the continuous data into 1 second windows with 50% overlap and marking those with excessive amplitude, kurtosis, improbable distributions, drift, or high-frequency muscle contamination. Marked segments were excluded from cleaning operations. Artifact attenuation was then performed with the sequential multi-channel Wiener filter (MWF) procedure. This included three passes targeting, in order: broadband muscle activity, eye blinks, and slow drift plus horizontal eye movements. After each pass, the corresponding artifact mask was used to construct spatio-temporal filters that reduced the targeted artifact while preserving neural activity [15]. Following MWF cleaning, the data were re-referenced to the robust common average after interpolation of removed channels. Independent component analysis (ICA) was performed using the extended Infomax algorithm on the cleaned, high-passed data [16]. ICA components were automatically classified using ICLabel, and those labelled as ocular, muscle, cardiac, line noise, or other non-brain sources were attenuated using wavelet-enhanced ICA (wICA), which reduces artifact variance while preserving potential neural signal within the component. Cleaned component activity was back-projected to the channel space. All previously removed channels were then re-interpolated to reconstruct the complete montage. The cleaned continuous data were then segmented into 2 sec epochs with 50% overlap [17]. Epochs spanning discontinuities were automatically rejected. Only artifact-free, wakeful epochs were retained for spectral and connectivity analyses.

Across groups, the number of retained artifact-free epochs and interpolated channels was comparable. In AD patients, a median of 289.5 (IQR 279.0-291.5) epochs were available with 2.0 (1.0-3.0) interpolated channels; in FTD, 266.0 (149.8-290.0) epochs with 1.0 (1.0-3.0) interpolated channels; in DLB, 330.5 (295.0-349.0) epochs with 3.0 (1.0-5.0) interpolated channels; and in healthy controls, 255.5 (144.5-292.5) epochs with 1.0 (0.0-1.0) interpolated channels.

## ***Spectral analysis***

Sensor-level power spectral density (PSD) was estimated using Welch’s method (EEGLAB spectopo) with window length set to the nearest power-of-two samples relative to the sampling rate and 50% overlap [18]. PSDs were computed over the 2-50 Hz range on concatenated artifact-free epochs for each electrode and converted from dB to absolute power. Relative band power was calculated per electrode for delta (2–4 Hz), theta (4–8 Hz), alpha 1 (8–10.5 Hz), alpha 2 (10.5–13 Hz), beta 1 (13–20 Hz), beta 2 (20–30 Hz), and gamma (30–50 Hz) bands by dividing band-specific power by the total 2–50 Hz power. Group-level topographies were generated by averaging relative power across participants within each group and visualized using EEGLAB topoplot with a fixed colour scale across groups within each frequency band.

## ***Source reconstruction and connectivity analysis***

Source-level analysis used a standard boundary element model (BEM) for forward modelling and the Schaefer-2018 cortical parcellation (100 parcels organized into seven large-scale networks: visual, somatomotor, dorsal attention, salience/ventral attention, limbic, control, default) [19]. For each frequency band, a linearly constrained minimum variance (LCMV) beamformer was computed for each participant to project cleaned sensor-level data into source space [20]. From the source-reconstructed time series, orthogonalized amplitude-envelope correlation (AEC) was computed between all parcel pairs. AEC is an amplitude-based metric that measures slow covariations in band-limited power after pairwise orthogonalization to suppress zero-lag correlations arising from volume conduction and spatial leakage. Connectivity spectra were estimated using multitaper DPSS windows with ±2 Hz smoothing (FieldTrip, tapsmofrq = 2) and 0.5 Hz frequency resolution, then averaged within each canonical frequency band (delta, 2–4 Hz; theta, 4–8 Hz; alpha, 8–13 Hz; beta, 13–30 Hz; gamma, 30–50 Hz) to yield one connectivity matrix per participant.

For visualization and network-level statistical analysis, participant-level matrices were reordered according to the Schaefer seven-network organization (Visual, Somato-Motor, Dorsal Attention, Salience/Ventral Attention, Limbic, Control, Default). Network-level connectivity was summarized by averaging parcel-wise AEC values within each network pair, producing 7×7 matrices per participant and frequency band. Diagonal elements of these matrices represent within-network connectivity strength, while off-diagonal elements represent between-network connectivity.

***Statistical analysis***

Demographic and clinical comparisons. Group comparisons of baseline clinical and demographic variables were performed using the Kruskal-Wallis *H* test for continuous variables or Fisher's exact test for categorical variables, as appropriate.

Hierarchical statistical framework. A hierarchical statistical approach was employed for all EEG analyses to control for potential confounders and multiple comparisons while maintaining sensitivity to true group differences. This framework consists of two stages: (1) omnibus testing to establish whether a significant group effect exists after adjusting for covariates, followed by (2) *post-hoc* pairwise comparisons conducted only at locations showing significant omnibus effects.

Stage 1: Omnibus testing. For each electrode (spectral power) or network pair (connectivity), a permutation-based one-way ANOVA was performed to test for group differences (HC, AD, FTLD, DLB) while adjusting for age, sex, and disease severity as covariates. The F-statistic for the group factor was computed from a general linear model (GLM): Y = β₀ + β₁·Group + β₂·Age + β₃·Sex + β₄·DiseaseSeverity + ε. Statistical significance was determined using Freedman-Lane permutation of residuals (5,000 permutations), which preserves covariate effects under the null hypothesis by: (1) fitting the reduced model (covariates only), (2) computing residuals, (3) permuting the residuals, (4) adding permuted residuals to fitted values from the reduced model, (5) fitting the full model to the permuted data, and (6) computing the F-statistic. This procedure was repeated 5,000 times to construct the null distribution. Effect size was quantified using partial eta-squared: η²p = SS_group / (SS_group + SS_error) where SS_group is the sum of squares attributable to the group factor and SS_error is the residual sum of squares. Effect sizes were interpreted as: small (0.01 ≤ η²p < 0.06), medium (0.06 ≤ η²p < 0.14), or large (η²p ≥ 0.14). Omnibus p-values were corrected for multiple comparisons using Benjamini-Hochberg FDR (α = 0.05) within each frequency band.

Stage 2: Post-hoc pairwise comparisons. For electrodes or network pairs showing significant omnibus effects (FDR-corrected p < 0.05), six pairwise comparisons were conducted: AD vs HC, FTLD vs HC, DLB vs HC, AD vs FTLD, AD vs DLB, and FTLD vs DLB. Each comparison used a permutation-based two-sample t-test (GLM contrast) with the same covariates (age, sex, disease severity) and Freedman-Lane permutation (5,000 permutations). Effect direction was quantified as t-values, with positive values indicating higher values in the first group of each contrast. Within each frequency band, pairwise p-values were corrected using Benjamini-Hochberg FDR (α = 0.05) across all tested locations and all six contrasts simultaneously.

Sensor-level spectral power. For each of the seven frequency bands (delta, theta, alpha 1, alpha 2, beta 1, beta 2, gamma), the hierarchical framework was applied across all 64 electrodes. Omnibus testing identified electrodes with significant group effects; *post-hoc* pairwise comparisons were then performed only at those electrodes. Results are displayed as scalp topographies showing t-values from pairwise comparisons (blue-white-red colormap) and η²p effect sizes from omnibus tests (white-purple colormap). Electrodes surviving FDR correction are marked with asterisks.

Network-level connectivity. For each of the five frequency bands (delta, theta, alpha, beta, gamma), the hierarchical framework was applied to the 28 unique network pairs (including 7 within-network connections on the diagonal) of the 7×7 connectivity matrices. Omnibus testing identified network pairs with significant group effects; *post-hoc* pairwise comparisons were then performed only at those pairs. Results are displayed as 7×7 heatmaps showing t-values from pairwise comparisons and η²p effect sizes from omnibus tests. Network pairs surviving FDR correction are shown with saturated colours; non-significant pairs are shown with pale/desaturated colours.

Visualization. For pairwise comparisons, a diverging blue-white-red colormap was used, with red indicating higher values in the first group (e.g., AD > HC) and blue indicating lower values. For omnibus effect sizes, a sequential white-purple colormap was used, with darker purple indicating larger η²p values. Colour scales were set adaptively within each frequency band to maximize visual contrast while maintaining interpretability.

All analyses were implemented in MATLAB R2024b using FieldTrip functions and custom scripts.

**Results**

**Table 1. Demographic and clinical features of included participants**

|  | **HC (n=32)** | **AD (n=56)** | **FTD (n=59)** | **DLB (n=26)** |
| --- | --- | --- | --- | --- |
| Age, yrs | 64.0 (55.3-67.6)^§^ | 69.1 (62.9-72.9)^§^ | 69.0 (58.1-73.1)^§^ | 75.6 (69.6-78.1)^*†‡^ |
| Sex, males (%) | 14 (43.8%) | 30 (53.6%) | 33 (55.9%) | 14 (53.8%) |
| Education | 13.0 (8.0-14.5)^§^ | 11.5 (8.0-13.0)^§^ | 11.0 (13.0-8.0)^§^ | 8.0 (6.5-11.0)^*†‡^ |
| Global CDR | - | 0.5 (0.5-0.5)^§^ | - | 0.5 (0.5-1.0)^‡^ |
| Global CDR plus NACC FTLD | - | - | 0.5 (0.5-0.5) | - |
| MMSE | 30.0 (30.0-30.0)^§†‡^ | 24.0 (23.0-25.0)^*^ | 23.0 (19.0-27.0)^*^ | 25.0 (22.0-26.0)^*^ |

Data are median (IQR) or n (%). n = number of participants; yrs = years; Global CDR = Global clinical dementia rating; Global CDR plus NAAC FTLD = Global CDR plus National Alzheimer’s Coordinating Center behavior and language domains; MMSE = mini-mental state examination; HC = healthy controls; FTD = frontotemporal dementia; AD = Alzheimer’s disease; DLB = dementia with Lewy bodies.

**P*<0.05 vs HC, †*P*<0.05 vs FTLD, ‡*P*<0.05 vs AD, §*P*<0.05 vs DLB, pairwise comparisons after significant interaction at the Kruskal-Wallis *H* test or at the Fisher’s exact test.

**Supplementary Figure 1.** Sensor-level relative power topographies in FTLD variants.

Grand-average scalp maps of relative EEG power for healthy controls (HC), primary progressive aphasia (PPA), and behavioural-variant frontotemporal dementia (bvFTD). Rows show canonical frequency bands: delta (2-4 Hz), theta (4-8 Hz), alpha 1 (8-10.5 Hz), alpha 2 (10.5-13 Hz), beta 1 (13-20 Hz), beta 2 (20-30 Hz), and gamma (30-50 Hz). Columns show groups. Warm colours indicate higher relative power, cool colours lower power. Asterisks mark electrodes that differ significantly from HC within the corresponding band (two-sided permutation GLM with age, sex, and disease severity as covariates; 5,000 permutations; Benjamini-Hochberg FDR q < 0.05); colour bars show t-values.

**References**

[1] McKhann GM, Knopman DS, Chertkow H, Hyman BT, Jack CR, Kawas CH, et al. The diagnosis of dementia due to Alzheimer’s disease: Recommendations from the National Institute on Aging-Alzheimer’s Association workgroups on diagnostic guidelines for Alzheimer’s disease. Alzheimer’s and Dementia 2011;7:263–9. https://doi.org/10.1016/j.jalz.2011.03.005.

[2] Rascovsky K, Hodges JR, Knopman D, Mendez MF, Kramer JH, Neuhaus J, et al. Sensitivity of revised diagnostic criteria for the behavioural variant of frontotemporal dementia. Brain 2011;134:2456–77. https://doi.org/10.1093/brain/awr179.

[3] Gorno-Tempini ML, Hillis AE, Weintraub S, Kertesz A, Mendez M, Cappa SF, et al. Classification of primary progressive aphasia and its variants. Neurology 2011;76:1006–14.

[4] McKeith IG, Dickson DW, Lowe J, Emre M, O’Brien JT, Feldman H, et al. Diagnosis and management of dementia with Lewy bodies Third report of the DLB consortium. Neurology 2005;65:1863–72.

[5] Fornari C, Mori F, Zoppi N, Libri I, Silvestri C, Cosseddu M, et al. Diagnostic Accuracy of the Five-Word Test for Mild Cognitive Impairment Due to Alzheimer’s Disease. Neurol Int 2022;14:357–67. https://doi.org/10.3390/neurolint14020029.

[6] Morris JC. The Clinical Dementia Rating (CDR): current version and scoring rules. Neurology 1993;43:2412–4. https://doi.org/10.1212/wnl.43.11.2412-a.

[7] Knopman DS, Kramer JH, Boeve BF, Caselli RJ, Graff-Radford NR, Mendez MF, et al. Development of methodology for conducting clinical trials in frontotemporal lobar degeneration. Brain 2008;131:2957–68. https://doi.org/10.1093/brain/awn234.

[8] Delorme A, Makeig S. EEGLAB: an open source toolbox for analysis of single-trial EEG dynamics including independent component analysis. J Neurosci Methods 2004;134:9–21.

[9] Oostenveld R, Fries P, Maris E, Schoffelen J-M. FieldTrip: Open Source Software for Advanced Analysis of MEG, EEG, and Invasive Electrophysiological Data. Comput Intell Neurosci 2011;2011:1–9.

[10] Rubinov M, Sporns O. Complex network measures of brain connectivity: Uses and interpretations. Neuroimage 2010;52:1059–69. https://doi.org/10.1016/j.neuroimage.2009.10.003.

[11] Bailey NW, Biabani M, Hill AT, Miljevic A, Rogasch NC, McQueen B, et al. Introducing RELAX: An automated pre-processing pipeline for cleaning EEG data - Part 1: Algorithm and application to oscillations. Clinical Neurophysiology 2023;149:178–201. https://doi.org/10.1016/j.clinph.2023.01.017.

[12] Gil Ávila C, Bott FS, Tiemann L, Hohn VD, May ES, Nickel MM, et al. DISCOVER-EEG: an open, fully automated EEG pipeline for biomarker discovery in clinical neuroscience. Sci Data 2023;10. https://doi.org/10.1038/s41597-023-02525-0.

[13] Klug M, Kloosterman NA. Zapline-plus: A Zapline extension for automatic and adaptive removal of frequency-specific noise artifacts in M/EEG. Hum Brain Mapp 2022;43:2743–58. https://doi.org/10.1002/hbm.25832.

[14] Bigdely-Shamlo N, Mullen T, Kothe C, Su KM, Robbins KA. The PREP pipeline: Standardized preprocessing for large-scale EEG analysis. Front Neuroinform 2015;9:1–19. https://doi.org/10.3389/fninf.2015.00016.

[15] Somers B, Francart T, Bertrand A. A generic EEG artifact removal algorithm based on the multi-channel Wiener filter. J Neural Eng 2018;15:036007. https://doi.org/10.1088/1741-2552/aaac92.

[16] Castellanos NP, Makarov VA. Recovering EEG brain signals: Artifact suppression with wavelet enhanced independent component analysis. J Neurosci Methods 2006;158:300–12. https://doi.org/10.1016/j.jneumeth.2006.05.033.

[17] Fraschini M, Demuru M, Crobe A, Marrosu F, Stam CJ, Hillebrand A. The effect of epoch length on estimated EEG functional connectivity and brain network organisation. J Neural Eng 2016;13. https://doi.org/10.1088/1741-2560/13/3/036015.

[18] Welch P. The use of fast Fourier transform for the estimation of power spectra: A method based on time averaging over short, modified periodograms. IEEE Transactions on Audio and Electroacoustics 1967;15:70–3. https://doi.org/10.1109/TAU.1967.1161901.

[19] Schaefer A, Kong R, Gordon EM, Laumann TO, Zuo X-N, Holmes AJ, et al. Local-Global Parcellation of the Human Cerebral Cortex from Intrinsic Functional Connectivity MRI. Cerebral Cortex 2018;28:3095–114. https://doi.org/10.1093/cercor/bhx179.

[20] Westner BU, Dalal SS, Gramfort A, Litvak V, Mosher JC, Oostenveld R, et al. A unified view on beamformers for M/EEG source reconstruction. Neuroimage 2022;246. https://doi.org/10.1016/j.neuroimage.2021.118789.
